# Effects of functional limitations and activities of daily living on the mortality of the older people: A cohort study in China

**DOI:** 10.3389/fpubh.2022.1098794

**Published:** 2023-01-20

**Authors:** Yumeng Gao, Liang Du, Jianping Cai, Tingfa Hu

**Affiliations:** ^1^Department of Medical Insurance, Jinshan Hospital of Fudan University, Shanghai, China; ^2^School of Public Health, Fudan University, Shanghai, China

**Keywords:** functional limitations, BADL, IADL, mortality, elderly, China

## Abstract

**Introduction:**

Prevalence of functional limitations (FLs) and disabled activities of daily living (ADL) cause heavy burdens to the health of the older people. Stratified by gender, this study aimed to explore the effects of FL and ADL on the mortality of the older people in China, and the mechanism was then discussed.

**Methods:**

We used survey data of a prospective 10-year cohort (2008–2018), from the China Longitudinal Healthy Longevity Survey (CLHLS). The primary outcome was all-cause mortality, and Independent variables included FL, basic ADL (BADL), and instrumental ADL (IADL). Covariates involved socio-demographic characteristics, health-related behaviors, and health status of the participants in the CLHLS project.

**Results:**

There were 967 (19.20%) male and 2,235 (32.36%) female older people performed functional limitations, and their survival time was 60.98 (SE = 0.66) and 55.19 (SE = 0.55) months, respectively. Old adults with FL had significantly poorer survival than the ones without (Log-rank test, *P* < 0.001). Weibull regression suggested that FL (*P* < 0.001), abnormal BADL (*P* < 0.001) and IADL (*P* < 0.001) were negatively associated with the survival of the older people. Further analysis showed that BADL and IADL performed significantly mediating roles in the relation of FL and survival time in old adults; additionally, for female older people, BADL also exhibited a significant moderating role in the effect of FL on survival.

**Conclusions:**

Prevalence of FL was serious among the older people in China, especially for the women. Disabilities of BADL and IADL and FL were negatively associated with the survival time of the older people in China. Regarding the effect of FL on survival, BADL and IADL performed significantly mediating roles, and the moderating role of BADL existed only for the female. These suggested evidence to implement strategies to maintain health in the older people.

## 1. Introduction

The trend of population aging has become one of the severe challenges in most countries, including China which has the largest older people population globally and is increasing rapidly (1). In China, the population over 60 years old accounted for 17.9% of the total population (1.4 billion), and the population over 65 years old accounted for 10.9%. With the population of older people growth, lots of public health problems are becoming increasingly prominent, such as supporting and care them, diseases prevention and treatment. Physically functional limitations (FLs), which commonly bring a high burden on the health-related quality of life among the older people, can be recognized as a matter of great concern in China.

FL means the ability to move or the physical movement of any body part being limited for some reasons, which is commonly measured by extremity functional limitation in practice (2–4). The older people with FL are often linked to higher utilization of healthcare, lower levels of social cohesion, and poor mental health status (4, 5). Suffering from FL, the older people are more likely to depend on caregivers to satisfy their life needs, which require assistance or adaptive equipment, in general, to improve mobility. Besides, serious FL of the older people can lead to various physical illnesses. Associations between chronic diseases and FL in the older people have been found for arthritis, diabetes, hypertension, coronary heart disease, cardiovascular disease, depression, stroke, and visual impairment (6). Additionally, these non-communicable diseases could then harm the quality of life of the older people. Therefore, we hypothesize that:

*H1: FL is directly associated with the death of the older people*.

Generally, ADL is composed of basic activities of daily living (BADL) and instrumental activities of daily living (IADL). BADL refers to the physically self-care ability (e.g., dressing), while IADL means the higher-level skills that the older people need to live independently in their communities (e.g., shopping). Previous studies have proven that ADL impairments are associated with increased mortality risk in old adults (7, 8). However, it still lacks concrete evidence whether disabled ADL associates with the death of the older people in China. Disabilities of ADL, which is highly prevalent in China, can cause many adverse consequences, such as poor physical or mental health, and an increased risk of accident injuries (9, 10). Besides, ADL disabilities reflect the fact that the older people have lost some of their self-care abilities, and to some extent, they also isolate the social interaction, which may augment the risk of mortality in an older population. Therefore, we hypothesize that:

*H2: disabled ADL is directly associated with the death of the older people*.

Previous studies reported that disability of ADL was triggered by a reduction in muscle strength in the lower limbs, with consequent difficulties in bathing, walking, toileting, and transferring, followed later by disability regarding dressing and eating (11, 12). However, to date, few researches have focused on the relation between ADL and FL of the older people in China. Because of FL, the older people may lose the ability to take care of themselves, like dressing, eating, and bathing; additionally, the instrumental activities of the older people can also be impeded, such as shopping, washing clothes, and taking public transportation. Hence, we speculate that FL is associated with ADL of the older people. Social participation of the older people is thought to be a vital issue in their active aging. Mobility of the older people with FL could be restricted, which may reduce their engagement in self-care and social activities, and then cause extensive damage to cognitive function and health beliefs with age; and ability of IADL and BADL continually weaken for the negatively cumulative effects from FL, which may result in an increased risk of death in old adults (13–15). Given these, we further hypothesized that:

*H3a: BADL mediates the relation between FL and death of the older people*.
*H3b: IADL mediates the relation between FL and death of the older people*.

Performance of ADL is an essential indicator of the health status of older adults. Influenced by traditional Chinese concepts and cultural consciousness, combined with underdeveloped healthcare system, home-based care has become the preferred way for older adults, so most of the older people with ADL disability have no access to professional care (16). The older people with BADL disability are often unable to undertake self-care in daily life, and if they have inadequate external care and support, there will be great possibilities that the negative events leading to death due to limited physical functions (16–18). Likewise, the old adults suffering from IADL are also restricted from engaging in social interaction and require indispensable assistance to maintain health, which could result in senior citizens with FL at high-risk mortality (19, 20). Based on the reasoning above, we hypothesize that:

*H4a: BADL moderates the relation between FL and death of the older people*.
*H4b: IADL moderates the relation between FL and death of the older people*.

**Figure 1:**
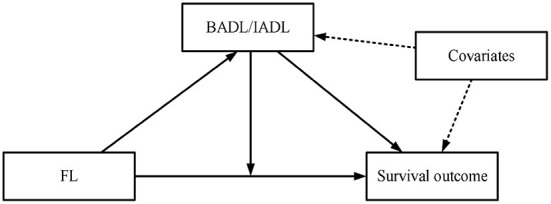
Potential study hypothesis based on literature review.

By literature review, it's noted that there are gender differences in both ADL disabilities (21, 22) and mortality (23) among older adults, and it is necessary to examine the hypotheses in male and female old adults, separately. Based on 10-years longitudinal data, stratified by gender, the purpose of this study was to examine the effects of FL and ADL on mortality in Chinese old adults. Furthermore, we explored the mediating and moderating effects of ADL on the relation between FL and mortality (Figure 1), which would provide a theoretical basis for promoting healthy aging in China in the future.

## 2. Methods

### 2.1. Study design and participants

The data used in this study came from the Chinese Longitudinal Healthy Longevity Survey (CLHLS), which is conducting by the Center for Healthy Aging and Development Studies, Peking University. The CLHLS is a nationwide survey that selects randomly half of the cities and counties in 23 provinces of China since 1998. This area had a total population of 1,156 million in 2010, accounting for approximately 85% of the Chinese population. The CLHLS collected information on the health status and quality of life of the participants who were aged 65 years and older.

The CLHLS datasets from the sixth wave (2008/2009, at baseline) to the ninth wave (2017/2018) were used in this study. Some countries consider that the older people should be aged 60 and above, while others consider it to be aged 65 and above. In order to better generalize the study conclusions to a wider population, this study was conducted for people aged 65 years and older. There were 16,954 participants at baseline, and we excluded 391 participants aged <65 years and 4,455 observations due to loss to follow-up, which led to 12,108 eligible participants in this study.

### 2.2. Variables and measurements

#### 2.2.1. Outcome definition

The primary outcome of this study was all-cause mortality. Mortality information was obtained from the follow-up survey done in 2011, 2014, and 2018. The date of death would be validated by death certificates when available–otherwise, the close family member's report was recorded. We then calculated the survival duration by months, from the investigation date to the participant's death.

#### 2.2.2. Independent variables

Functional limitations were measured by five objective movements' examinations, including hand behind neck, hand behind the lower back, raising arms upright, standing up from sitting in a chair, and pick up a book from the floor. These items have been proven to measure functional limitations validly in previous studies (24, 25). We scored each term as 0 if the participant could complete it without help, otherwise scored 1. The participant was identified as absent function-limitation if one can complete all the items without any help. IADL status was measured by a valid scale made by Lawton and Brody (26), which was consisted of eight items: visiting neighbors, shopping, cooking, washing clothes, walking one kilometer, carrying a five-kilogram object, crouching and standing three times, and taking public transportation. The response for each question was “yes”, “a little difficult” or “unable to do so”. We categorized respondents as IADL disabled (coded as 1) if they had any difficulty in performing these items (otherwise coded as 0). The basic activities of daily living (BADL) were measured using the Katz Index by six items: bathing, dressing, toileting, indoor transferring, eating, and continence (27). Disability in BADL was defined if the participants could not independently complete any above item. Response categories for BADL were consistent with IADLs and coded similarly.

#### 2.2.3. Covariates

This study covered three sets of potential confounders, including socio-demographic characteristics, health behaviors, and health status. The socio-demographic characteristics involved gender (male/female), age, education (schooling years), marriage status (married/unmarried/widowed), and household income (<10,000/10,000–30,000/30,001–60,000/>60,000 yuan per year). The health behaviors included smoking (yes/no), drinking (yes/no), and frequent physical exercise (yes/no). The health status was composed of body mass index (BMI), times of serious illness in the past 2 years, and the number of chronic diseases. BMI was calculated by [weight (in kilograms)/height (in meters) 2]. Several chronic diseases were measured by asking the question “Do you suffer from the following diseases?” The respondents could choose from 22 options, such as hypertension, diabetes, and stroke.

### 2.3. Statistical analysis

All analyses were stratified by gender, including subgroups of male, female, and total participants. Mean ± standard deviation (SD) was employed to describe the variables of normal distribution. Frequency and percentage (%) were applied to describe the categorical variables.

According to whether the older people had functional limitations or not, we calculated the means and its 95% confidence intervals (CIs) of survival time. Additionally, Kaplan-Meier survival analyses were used to depict the survival over time, and its 95% CI was estimated in the graph. Log-rank test was used to examine the equality of survival time in old adults with/without functional limitations. Then, adjusting three-sets covariates (socio-demographic characteristics, health behaviors, and health status), we employed Weibull regression to explore the risk factors of mortality in old adults, and regression coefficients and its standard error (SE) were reported. Furthermore, we tested the statistical assumption of the Weibull regression, and it was supported.

Finally, we explored the mediating and moderating effects of ADL (i.e., BADL & IADL) in the relation between functional limitations and mortality, stratifying by gender. Adjusting the covariates, we used the developed med4way command, in Stata software (Stata Corp LP, College Station, TX, USA), to calculate the mediating and moderating effects in this study. Med4way can be used when the outcome is continuous, dichotomous, count or survival time, and the mediator is continuous or binary. In this study, the outcome variables were survival data that did not satisfy the PH hypothesis after statistical testing (excluding Cox regression), and the probability of death increased rapidly over time (non-linear), so we employed the Weibull regression of accelerated failure-time form to explored the determinants of survival time of the older people. Additionally, due to binary BADL/IADL, Logistic regression was used for the mediator. Furthermore, med4way can decompose the overall effect of an exposure on an outcome into four components: (i) neither mediation nor interaction; (ii) just interaction (but not mediation); (iii) both mediation and interaction; and (iv) just mediation (but not interaction). Considering this study purpose, we only reported the mediation and interaction effects in the results. A detailed introduction of Med4way can be found elsewhere (28).

All the data analysis was done in the Stata 14.0 MP version (Stata Corp LP, College Station, TX, USA). All tests were two-sided, and *P* < 0.05 was considered statistical significance.

## 3. Results

### 3.1. Characteristics of the older people at baseline

We included 12,108 old adults at baseline. There were 7,009 (57.89%) female and 5,099 (42.11%) male older people. The average age of women (90.39 ± 11.18) was higher than that of men (85.07 ± 10.34). The total illiteracy rate was 65.63%, of which the female accounted for 74.96%. The divorced or widowed rate (71.84%) was high, and the rate of women (68.11%) was about twice as high as that of men (31.89%). About a half with an annual income of <10,000 yuan, while only 2,142 (17.69%) reported more than 30,000 yuan per year. Both smoking and alcohol consumption rates appeared higher among the male. Most of the older people (75.28%) lacked physical activities, and the female had a proportion of 62.21%. Mean BMI was 20.11 (SD = 3.56) in old adults, and most of the older people (82.72%) experienced no serious illness in the past 2 years, yet 2,092 (17.28%) did. Only 5,376 (44.40%) had no chronic diseases, but 2,917 (24.09%) had more than one chronic diseases; obviously, the female showed a higher rate of chronic diseases than the male ones (Table 1).

**Table 1:** Characteristics of the older people of a different gender at baseline.

**Variables**	**Total**	**Male**	**Female**
	**N (%)/mean** ±**SD**	**N (%)/mean** ±**SD**	**N (%)/mean** ±**SD**
Age, mean ± SD	88.15 ± 11.15	85.07 ± 10.34	90.39 ± 11.18
**Illiteracy**			
Yes	7,947 (65.63)	1,990 (25.04)	5,957 (74.96)
No	4,161 (34.37)	3,109 (74.72)	1,052 (25.28)
**Marriage status**			
Married	3,410 (28.16)	2,325 (68.18)	1,085 (31.82)
Unmarried/widowed	8,698 (71.84)	2,774 (31.89)	5,924 (68.11)
**Income (yuan/year)**			
<10,000	6,382 (52.71)	2,663 (41.73)	3,719 (58.27)
10,001–30,000	3,584 (29.60)	1,502 (41.91)	2,082 (58.09)
>30,000	2,142 (17.69)	934 (43.60)	1,208 (56.40)
**Smoking**			
Yes	2,091 (17.27)	1,741 (81.97)	377 (18.03)
No	10,017 (82.73)	3,385 (33.79)	6,632 (66.21)
**Drinking**			
Yes	2,116 (17.48)	1,496 (70.70)	620 (29.30)
No	9,992 (82.52)	3,603 (36.06)	6,389 (63.94)
**Physical activities**			
Yes	2,993 (24.72)	1,654 (55.26)	1,339 (44.74)
No	9,115 (75.28)	3,445 (37.79)	5,670 (62.21)
BMI, Mean ± SD	20.11 ± 3.56	20.58 ± 3.42	19.76 ± 3.62
**Serious illness in past two years**			
No	10,016 (82.72)	4,190 (41.83)	5,826 (58.17)
Yes	2,092 (17.28)	909 (43.45)	1,183 (56.55)
**Chronic diseases**			
No	5,376 (44.40)	2,241 (41.69)	3,135 (58.31)
One	3,815 (31.51)	1,607 (42.12)	2,208 (57.88)
Two or more	2,917 (24.09)	1,251 (42.89)	1,666 (57.11)

SD, standard deviation; BMI, body mass index.

### 3.2. Survival time of the older people in different gender

We calculated the survival time of the older people, and its difference was compared in the male and female, respectively. Survival time of the male and female older people were 60.98 (SE = 0.66) and 55.19 (SE = 0.55) months, respectively. In the male, there were 967 (19.20%) showed functional limitations, and their mean survival time was only 37.36 (SE = 1.17) months; while the older people without functional limitations showed a higher mean survival of 66.51 months (SE = 0.74). In the female, there were 2,235 (32.36%) showed functional limitations, and their mean survival time was only 36.35 (SE = 0.75) months; while the older people without functional limitations showed a higher mean survival of 64.20 months (SE = 0.69). Overall, there were 3,202 (26.81%) showed functional limitations, and their mean survival time was only 36.76 (SE = 0.63) months; while the older people without functional limitations showed a higher mean survival of 65.28 months (SE = 0.50) (Table 2).

**Table 2:** Survival months of the older people in different gender.

**Groups**	***N* (%)**	Mean^*^	**S.E**.	**95% CI**
**Male**				
Non-FL	4,069 (80.80)	66.51	0.74	(65.07, 67.96)
FL	967 (19.20)	37.36	1.17	(35.07, 39.65)
**Female**				
Non-FL	4,672 (67.64)	64.20	0.69	(62.85, 65.55)
FL	2,235 (32.36)	36.35	0.75	(34.89, 37.82)
**Total**				
Non-FL	8,741 (73.19)	65.28	0.50	(64.29, 66.26)
FL	3,202 (26.81)	36.76	0.63	(35.51, 38.00)

^*^largest observed analysis time is censored, mean is underestimated. FL, functional limitations; S.E., standard error; CI, confidence interval.

### 3.3. Kaplan-Meier survival estimates of the older people

**Figure 2:**
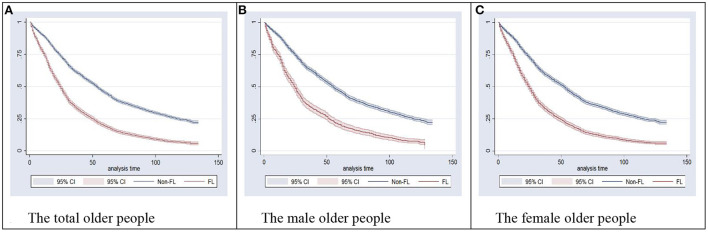
Kaplan-Meier survival estimates of the older people in different gender. **(A)** The total older people. **(B)** The male older people. **(C)** The female older people. Log-rank test was used to examine the equality of survivor functions among the total, male, and female older people, which indicated a significant difference between the FL and non-FL groups (*P* < 0.001). FL, functional limitations; CI, confidence interval.

Kaplan-Meier survival analysis was used to estimate the difference of mortality in the older people with different functional status. Overall, it indicated that old adults with functional limitations had poorer survival than the ones without, and this difference was statistically significant (Log-rank test, Chi-square = 1,133.05, *P* < 0.001). Additionally, it showed that, over time, old adults with functional limitations had significantly poorer survival than the ones without, for either the male (Log-rank test, Chi-square = 389.64, *P* < 0.001) or female (Log-rank test, Chi-square = 690.41, *P* < 0.001) older people in China (Figure 2).

### 3.4. Weibull regression for determinants of survival time

Weibull regression was used to explored the determinants of survival time in the older people. It suggested that functional limitations were negatively associated with the survival time of the older people (coefficient= −0.11, *P* < 0.001), and the male older people (coefficient = −0.16, *P* < 0.001) showed a higher risk of mortality than the female ones (coefficient = −0.11, *P* < 0.001). IADL disabilities also exhibited a significantly negative association with the survival time in old adults (coefficient = −0.25, *P* < 0.001). BADL disabilities negatively associated with the survival of the older people (coefficient = −0.27, *P* < 0.001), and the male (coefficient = −0.23, *P* < 0.001) showed a lower risk of mortality than the female (coefficient = −0.29, *P* < 0.001).

Besides, the risk of mortality in old adults (coefficient = −0.05, *P* < 0.001) increased with age. Comparing to the married older people, the unmarried/widowed showed a higher risk of mortality (coefficient = −0.11, *P* < 0.001), and the male (coefficient = −0.13, *P* < 0.001) showed a lower risk of mortality than the female ones (coefficient = −0.24, *P* < 0.001). No physical activities engagement exhibited a significant risk of mortality (coefficient = −0.10, *P* < 0.001). BMI exhibited positive effects on survival time (coefficient = 0.01, *P* < 0.01) (Table 3).

**Table 3:** Weibull regression for determinants of survival time of the older people.

**Variables**	**Total**		**Male**		**Female**	
	**Coefficient**	**S.E**.	**Coefficient**	**S.E**.	**Coefficient**	**S.E**.
FL	−0.11^***^	0.021	−0.16^***^	0.036	−0.11^***^	0.027
IADL disabilities	−0.25^***^	0.022	−0.27^***^	0.032	−0.25^***^	0.030
BADL disabilities	−0.27^***^	0.024	−0.23^***^	0.041	−0.29^***^	0.029
Age	−0.05^***^	0.001	−0.05^***^	0.002	−0.05^***^	0.001
Illiteracy	−0.09^***^	0.020	0.02	0.028	0.04	0.036
Unmarried/widowed	−0.11^***^	0.024	−0.13^***^	0.030	−0.24^***^	0.043
Income	0.01	0.011	0.01	0.018	−0.01	0.015
No smoking	0.12^***^	0.025	0.02	0.030	0.08	0.050
No drinking	0.02	0.024	−0.05	0.031	−0.01	0.040
No physical activities	−0.10^***^	0.021	−0.11^***^	0.030	−0.11^***^	0.031
BMI	0.01^***^	0.003	0.01^**^	0.004	0.02^***^	0.003
Serious illness experience	−0.07^**^	0.024	−0.07^*^	0.037	−0.05	0.032
Chronic diseases	−0.02	0.011	−0.04^*^	0.018	−0.01	0.015

^*^P < 0.05, ^**^P < 0.01, ^***^P < 0.001.

FL, functional limitations; BADL, basic activities of daily living; IADL, instrumental activities of daily living; BMI, body mass index; S.E., standard error.

### 3.5. Mediation and moderation role of ADL on survival

Finally, we explored the mediating and moderating role of IADL and BADL in the effect of functional limitations on survival time in the older people. For the male, the disabilities of BADL [coefficient = −0.06, 95% CI (−0.08, −0.04)] and IADL [coefficient = −0.08, 95% CI (−0.10, −0.06)] performed significantly mediating roles in the relation of functional limitations and survival time in old adults. For the female, the disabilities of BADL [coefficient = −0.11, 95% CI (−0.12, −0.09)] and IADL [coefficient= −0.08, 95% CI (−0.09, −0.06)] performed significantly mediating roles in the relation of functional limitations and survival in old adults; additionally, BADL [coefficient = −0.11, 95% CI (−0.18, −0.04)] exhibited a significantly moderating role in the effect of functional limitations on survival time. Overall, the disabilities of BADL [coefficient = −0.08, 95% CI (−0.10, −0.07)] and IADL [coefficient = −0.08, 95% CI (−0.10, −0.07)] performed significantly mediating roles in the relation of functional limitations and survival time in old adults; additionally, BADL [coefficient = −0.10, 95% CI (−0.14, −0.02)] exhibited a significantly moderating role in the effects of functional limitations on survival time (Table 4).

**Table 4:** Mediation and moderation role of ADL on survival of the older people.

**Specific effects**	**Coefficient**	**S.E**.	**Z**	***P-*value**	**95% CI**
**Male**					
FL → BADL → Survival	−0.06	0.01	−5.71	<0.001	(−0.08, −0.04)
FL × BADL → Survival	−0.02	0.06	−0.31	0.753	(−0.13, 0.09)
FL → IADL → Survival	−0.08	0.01	−7.44	<0.001	(−0.10, −0.06)
FL × IADL → Survival	0.04	0.05	0.94	0.346	(−0.05, 0.13)
**Female**					
FL → BADL → Survival	−0.11	0.01	−10.81	<0.001	(−0.12, −0.09)
FL × BADL → Survival	−0.11	0.04	−3.02	0.002	(−0.18, −0.04)
FL → IADL → Survival	−0.08	0.01	−8.17	<0.001	(−0.09, −0.06)
FL × IADL → Survival	0.001	0.03	0.02	0.985	(−0.06, 0.06)
**Total**					
FL → BADL → Survival	−0.08	0.01	−11.82	<0.001	(−0.10, −0.07)
FL × BADL → Survival	−0.08	0.03	−2.66	0.008	(−0.14, −0.02)
FL → IADL → Survival	−0.08	0.01	−10.99	<0.001	(−0.10, −0.07)
FL × IADL → Survival	0.007	0.03	0.28	0.780	(−0.04, 0.06)

FL, functional limitations; BADL, basic activities of daily living; IADL, instrumental activities of daily living; S.E., standard error; CI, confidence interval.

“ × ”meant the moderating effects, and → meant the regression path.

## 4. Discussion

Exploring the prevalence and determinants of survival status among the older people is the basis of public health policy formulation and implementation, such as allocation of resources and health service planning, and are important for the healthy aging process. To our knowledge, no study to date has examined the effects of FL and ADL on the mortality of the older people in China. Using a nationwide 10-year cohort, by gender stratification, this study has explored the potential mechanism regarding the effects of FL and ADL on survival in the older people, which provided evidence to implement strategies to maintain their health.

This study found that the prevalence of FL was serious among the older people in China, and about a quarter (26.81%) suffered from FL at baseline. FL is a common kind of physical frailty when people get older, while it always influences the physiological wellbeing, mental health, and social interaction of the older people (29, 30). Higher prevalence of functional limitations could be associated with the absence of physical activities in the older people, and this study showed that about three quarters of them lacking physical activities in daily life. Previous studies have proven that physical activities could improve cardiorespiratory fitness, muscular strength, body composition, balance, flexibility, and muscular endurance in the older people (31). Hence in the future, more access to physical exercise should be provided to the older people to strengthen their functional health. Additionally, this study showed that the prevalence of FL was higher in the female older people than the male, which was in line with previous studies (32, 33). This finding, in practice, can be interpreted for many reasons: including under-education, unfavorable biological characteristics, higher propensity to admit illness, and different social roles and relationships, which could make women more vulnerable to poor health (34). Furthermore, the results indicated that the older people without FL had about extra 29 survival months longer than those with FL. Analyzing the ten-year survival rate, we found the older people with FL was <10% but non-FL older people was more than 20%. However, at present, the problems of FL in old adults have not yet received much attention from the public. Therefore, effective interventions should be put forward and implemented to the prevalence of FL in China, which ensures healthy late life of the aged population.

This study showed that FL was negatively associated with the survival time of the older people, and the survival of the female was more damaged than the male. The older people with FL often perform various problems of physician-rated health, and this could increase their risks of mortality (35). Additionally, FL could arise mental distress, such as anxiety, depression, and even suicidal thought, especially to the female, which multiply a negative impact on the survival of the older people (36). Also, ADL disabilities exhibited a significantly negative association with survival in old adults. IADLs are essential to living independently in a community, and IADL performance is thought to reflect underlying cognitive and physical function (37). So if an aged person is diagnosed with disabled IADL, then he/she could have a higher risk to die younger than the coeval. BADLs are the most important factors in characterizing the health status of frail old adults, and it is ordinarily evaluated to determine the levels of care that people should receive, which costs a lot in China (38). Low ADL level, causing greater negative impacts on women, has been proven to be associated with various chronic diseases, abnormal physiological indicators, and nutritional incidence in old adults, which can rise the possibility of mortality (21, 39). A population-based health survey in Norway found that physical inactivity could be the most important lifestyle risk factors for ADL/IADL disability (40). To compress the prevalence and harm of ADL disabilities of the older people in China advances in medications, healthy lifestyle, and socioeconomics should be achieved in the future (41).

This study found that the unmarried/widowed, comparing to the married older people, showed a higher risk of mortality, and the male showed a lower risk of mortality than the female ones. Family support networks, especially spousal support, and living arrangements are likely to be the most common determinants for the health of the older people in the current Chinese context (42). The married older people, especially the women, could have better health status than the unmarried ones because the spouses of the older people are the ones who spend the most lifetime with them, who have the most intimate relationship, and the loss of the spouse can spontaneously stimulate the mortality risk of the older people (43, 44). Besides, the results suggested that no physical activities engagement exhibited a significant risk of mortality in senior citizens. However, in daily life, a previous study indicated that over 50% of the older people do not meet recommended levels of regular physical activity (45), so we should take effective measures to improve the activities among old adults. Interestingly, our study also found that BMI exhibited a positive association with survival time. Higher BMI always implies a better living standard for the older people, so they are characterized by high survival duration.

Exploring the mediating and moderating role of ADL on the relation between FL and survival of the older people, we found that the disabilities of BADL negatively mediated the relation between FL and survival in old adults. This suggests that FL could increase the likelihood of incidence of BADL disability, and then to some extent, it increases the death of the older people. Therefore, improving the prevalence of FL in the older people cannot fundamentally prompt the survival increase, and further attention should be given to the impact of BADL on the survival of the older people in the future, to achieve better healthy aging in China (41). Additionally, we found that the disabilities of IADL negatively mediated the relation between FL and survival in old adults. The older people with FL were limited on physical activities, increasing the risk of disabled IADL, which greatly reduced the health status of the older people. IADL is an important indicator that affects health, but its impact on the health or death of the older people is often overlooked in China (9, 46). Hence, monitoring the IADL and FL condition of the older people to reduce the occurrence of adverse events is an important recommendation to improve their quality of life.

As the results showed, BADL disabilities negatively moderated the effect of FL on the survival of the older people. Impairment in the performance of BADL, especially in household settings, is considered far more complex than cellular or molecular mechanisms of aging due to the complex body and environmental systems involved (47). BADL disabilities often indicate a loss in muscle strength, which can jeopardize the health and even lead to mortality of the older people. Experiencing extreme difficulty in BADL and healthcare demand are not only related to decreased quality of life but also increased likelihood of long-term nursing home placement; and in this context, the older people with FL, generally accompanied by poor ability of balance mobility, could be suffering from higher risk of mortality (48). Stratified by gender, however, the results showed, for the male, BADL disabilities indicated no moderating effect on the relation between FL and survival. This may be since disability regarding ADL is more prevalent among the female, and BADL doesn't have that much impact on the lives of the male (21). Besides, the men older people perform lower frailty and better resilience than the women, which could interpret the no existence of moderating role of BADL in the male (21, 33, 49). Therefore, screening and improving the ADL, especially for the women, should arise wide public concern to develop the old-age policy in the future.

However, some limitations should be mentioned in this study. Firstly, FL and ADL (BADL/IADL), like many previous studies, were collected through self-report, which might lead to fuzzification of exposure. Secondly, more than a quarter of the cohort has been lost to follow-up, and most of the lost to follow-up were due to city construction and moving of living places. This may generate bias in our study results. Thirdly, since data from the CLHLS did not allow us to determine the reason for mortality, we thus did not further attribute FL and ADL disabilities to specific-cause mortality in the current study. Further studies focusing on the association of different subtypes and the duration of FL and ADL with mortality are needed. Finally, if we accounted for a set of common confounders, the results could be more robust. Some potential covariates, either unmeasured (e.g., medical treatment and diet) or unknown, might confound the association between FL, ADL, and mortality. The intensity and type of physical activities (strength training; walking; dancing; running; aquatic activities, etc.) could influence the ADL and survival status, while it was not collected in the CLHLS survey, which need explore the detailed associations among them in future study.

## 5. Conclusions

Prevalence of FL was serious among the older people in China, especially for the women, and the older people without FL had longer survival than that with FL. Disabilities of BADL and IADL and FL were negatively associated with the survival time of the older people in China. Additionally, the risk of mortality improved in old adults of higher age, women, unmarried/widowed marriage status, no physical activities engagement, and lower BMI. Disabilities of BADL and IADL performed significantly mediating roles in the relation of FL and survival. BADL disabilities negatively moderated the effect of FL on the survival of the older people, but this moderating effect was not significant for the male.

## Data availability statement

The datasets presented in this study can be found in online repositories. The names of the repository/repositories and accession number(s) can be found here: http://opendata.pku.edu.cn/dataverse/CHADS&lt.

## Ethics statement

The studies involving human participants were reviewed and approved by Duke University Health System Institutional Review Board. The patients/participants provided their written informed consent to participate in this study.

## Author contributions

YG contributed to the management of the whole study, including the study conception, design, analysis, and interpretation of data, and drafted the article. LD, JC, and TH contributed to the revision of the manuscript for important intellectual content. All authors contributed to the article and approved the submitted version.
